# Laser ablation for tumor ingrowth of the bridging stent via the endoscopic ultrasound-guided hepaticogastrostomy

**DOI:** 10.1055/a-2772-5660

**Published:** 2026-01-20

**Authors:** Yuichi Suzuki, Haruo Miwa, Kazuki Endo, Ritsuko Oishi, Hiromi Tsuchiya, Manabu Morimoto, Shin Maeda

**Affiliations:** 1 26437 Gastroenterological Center, Yokohama City University Medical Center, Yokohama, Japan; 2 26438 Department of Gastroenterology, Yokohama City University Graduate School of Medicine, Yokohama, Japan

Endoscopic ultrasound-guided hepaticogastrostomy (EUS-HGS) with bridging stenting is a valuable drainage option for malignant hilar biliary obstruction (MHBO) when endoscopic transpapillary drainage fails 1 2 3 . However, an optimal strategy for managing recurrent biliary obstruction after bridging stenting through the EUS-guided created route (ESCR) has not been established. Although transpapillary reintervention using laser ablation for self-expandable metallic stent (SEMS) dysfunction due to tumor ingrowth has been reported 4 , we report a first case of MHBO after pancreaticoduodenectomy in whom trans-ESCR laser ablation was successfully performed ( Video 1 ).

Laser ablation via the endoscopic ultrasound-guided created route offers a novel therapeutic option for reintervention in patients with malignant hilar biliary obstruction.Video 1

**Fig. 1:**
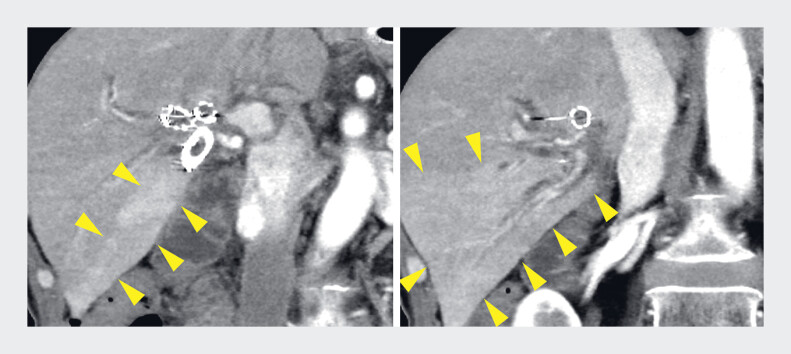
Contrast-enhanced computed tomography (CT) shows dilation of the B6 bile duct and increased enhancement of the posterior segment (arrowheads).

**Fig. 2:**
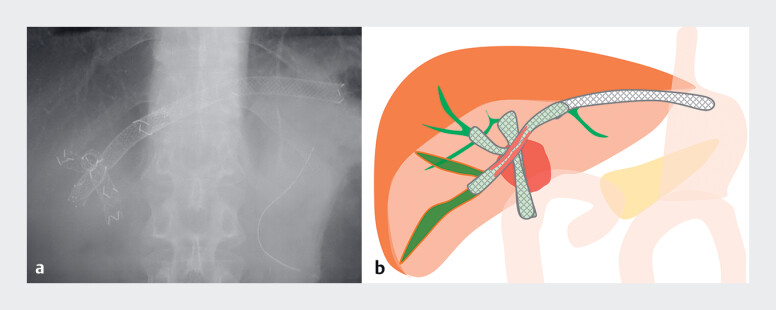
**a** A fluoroscopic image before the procedure shows two uncovered self-expandable metallic stents (UCSEMSs) in the anterior and posterior bile ducts, and the SEMSs of endoscopic ultrasound-guided hepaticogastrostomy with bridging stenting from the B2 to the isolated B6. **b** Schema illustrating tumor ingrowth into the bridging stent (the red-highlighted region within the stent).

**Fig. 3:**
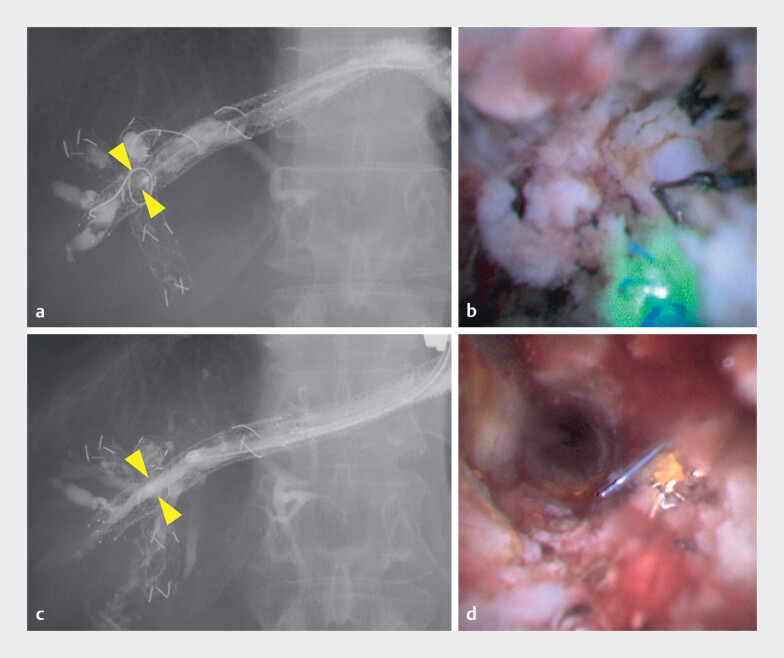
**a** Cholangiography before the procedure shows a stricture within the bridging stent (yellow arrowheads). **b** Cholangioscopy before the procedure shows tumor ingrowth through the stent mesh. **c** Cholangiography after the procedure shows improvement of the stricture (yellow arrowheads). **d** Cholangioscopy after the procedure shows recanalization of the bridging stent.

A 60-year-old man developed a Bismuth type IIIa stricture due to recurrent ampullary carcinoma after pancreaticoduodenectomy. Two uncovered SEMSs (UCSEMSs) were placed in the anterior and posterior bile ducts by balloon enteroscopy-assisted endoscopic retrograde cholangiopancreatography, and EUS-HGS with bridging stenting was performed from the B2 to the isolated B6. During ongoing chemotherapy, he was admitted for segmental cholangitis at B6 ( Fig. 1 and Fig. 2 ). Therefore, reintervention via the ESCR was performed ( Fig. 3 ).

Cholangiography revealed a stricture within the bridging stent due to tumor ingrowth. Subsequently, balloon dilation was performed for the stricture using a balloon catheter (REN 8-mm; Kaneka Corporation, Osaka, Japan), followed by the insertion of a cholangioscope (eyeMAX 9-Fr; Micro-Tech, Nanjing, China). Since cholangioscopy revealed tumor ingrowth through the stent mesh, laser ablation was performed using a Holmium:YAG laser (LithoEVO, Edap TMS, Lyon, France) with an energy setting of 0.8 J and a frequency of 12 Hz. After laser ablation, the SEMS lumen was successfully recanalized, as confirmed by both cholangioscopy and cholangiography. Therefore, additional stent placement was not required. He was discharged without any adverse events, and chemotherapy was subsequently resumed.

To the best of our knowledge, this is the first report of trans-ESCR laser ablation for tumor ingrowth, offering a novel therapeutic option for reintervention in patients with MHBO.

Endoscopy_UCTN_Code_TTT_1AR_2AF
